# Decreased Expression of Hsa_circ_00001649 in Gastric Cancer and Its Clinical Significance

**DOI:** 10.1155/2017/4587698

**Published:** 2017-01-12

**Authors:** Wen-han Li, Yong-chun Song, Hao Zhang, Zhang-jian Zhou, Xin Xie, Qing-nuo Zeng, Kun Guo, Ting Wang, Peng Xia, Dong-min Chang

**Affiliations:** ^1^The First Affiliated Hospital, Xi'an Jiaotong University, Xi'an, China; ^2^The Second Subsidiary Hospital, Fourth Military Medical University, Xi'an, China; ^3^The Second Affiliated Hospital, Xi'an Jiaotong University, Xi'an, China

## Abstract

*Background*. It has been reported that circRNAs are differentially expressed in a wide range of cancers and could be used as a new biomarker for diagnosis. However, the correlation between circRNAs and gastric cancer (GC) it is still unclear.* Materials and Methods*. In this study, by using real-time quantitative reverse transcription-polymerase chain reactions (qRT-PCRs), we detected the expression level of hsa_circ_0001649 in tissue and serum samples from GC patients.* Results*. We found that hsa_circ_0001649 expression was significantly downregulated in GC tissue compared with their paired paracancerous histological normal tissues (PCHNTs) (*P* < 0.01). We next analyzed the expression level of hsa_circ_0001649 in serum samples between preoperative and postoperative GC patients. We found that its level in serum was significantly upregulated after surgery (*P* < 0.01). The area under the receiver operating characteristic (ROC) curve was 0.834. Moreover, the expression level of hsa_circ_0001649 was significantly correlated with pathological differentiation (*P* = 0.039).* Conclusion*. Our test suggested that hsa_circ_0001649 was significantly downregulated in GC and may become a novel potential biomarker in the diagnosis of GC.

## 1. Introduction

Although the incidence of gastric cancer (GC) has declined in recent years, it is still one of the common malignancies worldwide, accounting for 841,000 deaths in 2013 [1]. Patients with advanced gastric cancer are often associated with extremely poor prognosis [2]. Therefore, developing a diagnosis panel in GC may help discovering the susceptible population earlier, which could increase the patients' probability to achieve complete cure.

Circular RNAs (circRNAs) are a large class of endogenous noncoding RNAs that attract increasing attention in the field of RNA recently. Compared with linear RNAs that are terminated with 5′caps and 3′tails, circRNAs exhibited a remarkable characteristic of undergoing “backsplicing” without a free 3′ or 5′ end [3–5]. Subsequent reports revealed that circRNAs participate in a wide range of biological processes, including competition with endogenous RNAs for binding to miRNAs, or regulate alternative splicing [4, 6]. It was also demonstrated that circRNAs are involved in the initiation and progression of several types of cancer [7–9]. Moreover, compared with mRNAs, circRNAs are resistant to exonucleolytic activities and are stable in extracellular space. Memczak et al. [10] found that circRNAs are readily detectable in clinical whole blood specimen. The above evidence led us to wonder whether circRNAs serve as a new biomarker for tumor diagnosis, prognosis, and therapeutic response prediction [11, 12].

By analyzing bioinformatics information in two circRNA databases (CircBase and circ2Traits), we predicted that hsa_circ_0001649, which is located at chr6:146209155-146216113, has a strong association with GC (*P* < 0.01). Its associated gene symbol is a tumor suppressor gene named SHPRH. Depletion of SHPRH could be observed in a variety of cancer types, such as prostate cancer, ovarian cancer, and liver cancer [13, 14]. In the present study, we set out to detect hsa_circ_0001649 in tissue and serum samples from patients harboring gastric tumor at various stages. Besides, we also analyzed the relationships between hsa_circ_0001649 expression level and clinicopathological findings to assess the diagnostic value of this marker for the early detection of primary GC.

## 2. Materials and Methods

### 2.1. Patients and Clinical Specimens

In order to reduce bias, we designed this experiment as a blinded assay. All samples' collection and preservation were done by a person who did not participate in the follow-up studies. Patients with primary GC who participated in this study were recruited consecutively. The study material of this study included 76 tumor tissue samples and their paired paracancerous histological normal tissues (PCHNTs) which were obtained during curative surgery. In the meantime, 20 patients' whole blood samples were collected preoperatively and postoperatively (more than 20 days after surgery). None of the experimental subjects had received prior gastric resection or preoperative chemotherapy/radiation therapy. All samples were immediately frozen and stored at −80°C until total RNA was extracted. In order to reduce bias, samples were randomly coded before processing. All patients voluntarily joined this study with written informed consent to have their biologic specimens analyzed. This study was announced by the Ethical Committee of the First Affiliated Hospital of Xi'an Jiaotong University.

### 2.2. Cell Culture

Immortalized human gastric cancer cell lines, SGC-7901, were used in this study. We purchased the cell line from the Type Culture Collection of the Chinese Academy of Sciences (Shanghai, China). All cells were routinely cultured in RPMI-1640 medium (Gibco) supplemented with 10% fetal bovine serum (Hyclone) at 37°C in a humidified atmosphere containing 5% CO_2_.

### 2.3. Total RNA Extraction

TRIzol reagent (Ambion, life technologies, USA) was used to extract RNA from cells and tissues according to the manufacturer's instructions, and total RNA in plasma was extracted using TRIzol LS Reagent (Invitrogen), following the manufacturer's instructions. Then, concentration of RNA was measured by ultraviolet spectrophotography.

### 2.4. Reverse Transcription

cDNA was synthesized by reverse transcription (RT) using a Primescript RT reagent kit with random primers according to manufacturer-provided protocols (TaKaRa).

### 2.5. Real-Time Quantitative Reverse Transcription-Polymerase Chain Reaction (qRT-PCR)

The qRT-PCR was achieved using SYBR Premix Ex Taq™ II (Tli RNaseH Plus) (TaKaRa) on CFX96 Real-Time PCR Detection System (Bio-Rad, California, USA) following the manufacturer's instructions. Divergent primers, rather than convergent primers, were synthesized by Sangon Biotech (Shanghai, China). We use GAPDH as an internal control. The primers used for qRT-PCR are summarized in Table 1. All reactions were performed in triplicate.

### 2.6. CEA, CA19-9, and CA-724 Measurements

Normal levels of CEA, CA19-9, and CA-724 were defined as <3.4 ng/mL, <39 U/mL, and <9.8 U/mL, respectively. The tests were done independently at the clinical laboratory in the First Affiliated Hospital of Xi'an Jiaotong University College of Medicine.

### 2.7. Statistical Analysis

Statistical analysis was performed with the SPSS 13.0 software (SPSS, Chicago, IL, USA) and GraphPad Prism 5.0 (GraphPad Software, La Jolla, CA). The qPCR results were analyzed using 2^−ΔΔCt^ method. The correlation of hsa_circ_0001649 expression level between GC and their matched gastric nontumorous tissues or serum samples were calculated using paired* t*-test. The correlations between hsa_circ_0001649 levels and clinicopathological factors were further analyzed by one-way analysis of variance (ANOVA). Receiver operating characteristic (ROC) curve was constructed using SPSS 13.0 to evaluate the diagnostic values. *P* values < 0.05 (two-sided) were considered statistically significant.

## 3. Results

### 3.1. Patient Characteristics

In order to explore the expression level of hsa_circ_0001649 in GC, 76 paired GC and PCHNTs tissue samples (including 61 males and 15 females) were enrolled in this study. The mean age of GC patients was 57.9 ± 11.6. Besides, we analyzed hsa_circ_0001649 expression level in 20 paired preoperative and postoperative serum samples.

### 3.2. Existence of Hsa_circ_0001649 in Gastric Cancer Cells

**Figure 1:**
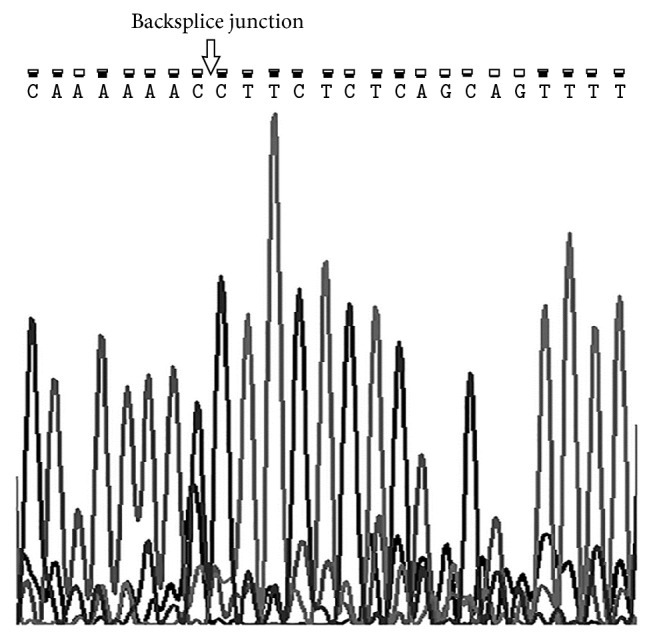
Sanger sequencing result of hsa_circ_0001649 showed the backsplice junction.

We used divergent primers to amplify hsa_circ_0001649 in SGC-7901 cell line. The amplified product yielded a single peak in a melting curve analysis. The qRT-PCR products were then sequenced and the result showed that the sequence was completely consistent with that from CircBase (Figure 1). So we concluded that hsa_circ_0001649 existed in gastric cancer and could be amplified by qRT-PCR.

### 3.3. Hsa_circ_0001649 Expression Was Downregulated in Gastric Cancer Tissues

**Figure 2:**
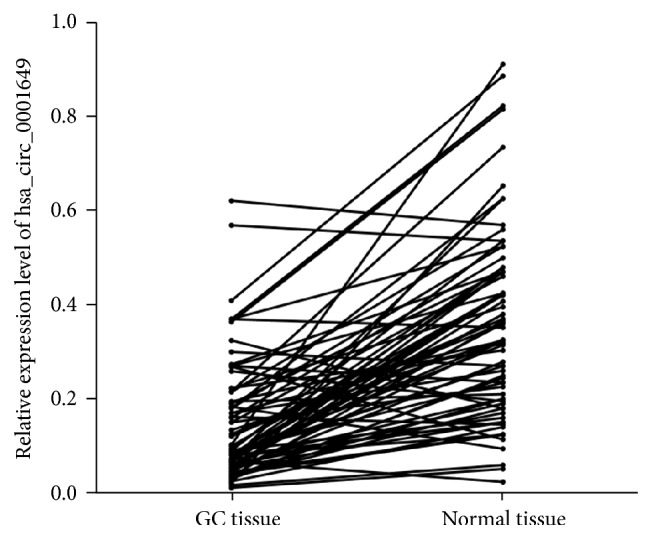
The expression levels of hsa_circ_0001649 in GC tissue samples and their paired PCHNTs. The picture showed that hsa_circ_0001649 expression in GC tissue was significantly lower than those in corresponding nontumorous tissues (*n* = 76, *P* < 0.01).

We first examined the expression level of hsa_circ_0001649 in 76 tissue samples of GC patients and PCHNTs by qRT-PCR, when using GAPDH as the internal standard. Our results showed that hsa_circ_0001649 expression in GC tissue was significantly lower than those in corresponding nontumorous tissues (*n* = 76, *P* < 0.01) (Figure 2).

### 3.4. Hsa_circ_0001649 Expression Was Upregulated in Gastric Cancer Serum Samples after Surgery

**Figure 3:**
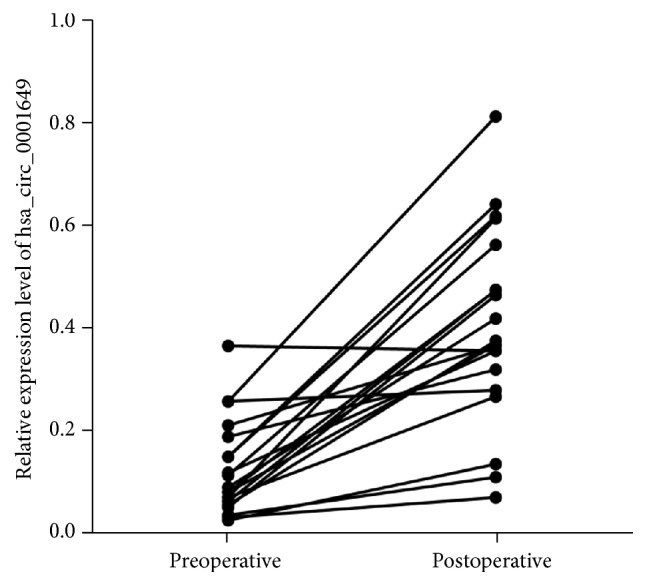
The expression levels of hsa_circ_0001649 in serum samples was significantly upregulated in those serum samples collected postoperatively (*n* = 20, *P* < 0.01).

To further investigate whether hsa_circ_0001649 could be used as a biomarker for GC, we detected its expression level in serum samples. Our data suggested that comparing with preoperatively collected samples, hsa_circ_0001649 expression level was significantly upregulated in those serum samples collected postoperatively (*P* < 0.01) (Figure 3). The above evidence suggested that hsa_circ_0001649 was downregulated in either GC tissue samples or GC serum samples compared to control groups (all *P* values < 0.01)and, therefore, may be considered as a panel for the early detection of GC.

### 3.5. Potential Diagnostic Values of Hsa_circ_0001649 in Gastric Cancer

**Figure 4:**
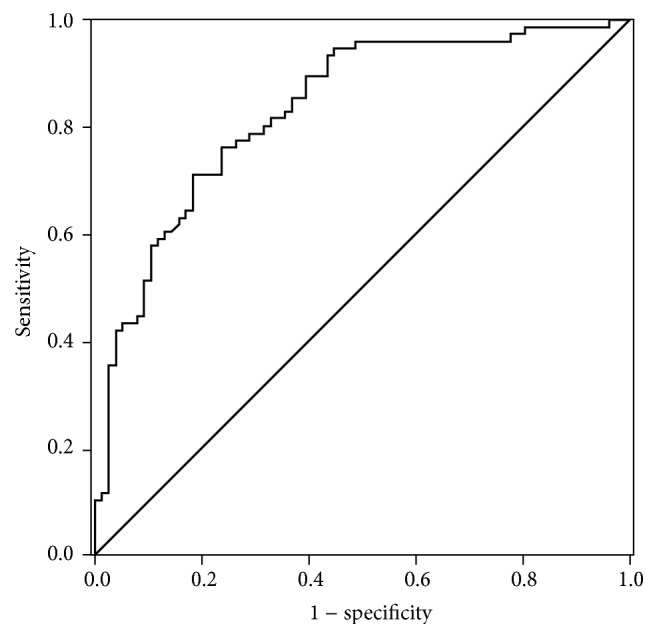
ROC curve of Hsa_circ_0001649. Area under the curve was 0.834. The sensitivity and specificity were 0.711 and 0.816, respectively.

Our results revealed differential expression of hsa_circ_0001649 between gastric cancer tissues and nontumorous tissues as well as in plasma samples. We next explored the correlation between clinicopathological data and the expression level of hsa_circ_0001649; the results were shown in Table 2. We found that hsa_circ_0001649 expression level was associated with pathological differentiation (*P* = 0.039). On the contrary, no correlation was found of hsa_circ_0001649 expression level with other clinicopathological factors, including age, gender, TNM stage, lymphatic metastasis, CEA, CA19-9, and CA-724 levels. Then we built a ROC curve to estimate the diagnostic values of this circRNA in gastric cancer. The sensitivity and specificity were 0.711 and 0.816, respectively. The cutoff value was 0.22692250 and the area under the curve was 0.834 (Figure 4).

## 4. Discussion

circRNAs are novel members of noncoding RNA family that are formed by the noncanonical splicing of linear pre-mRNAs. Although circRNAs have been known to exist for 20 years [15–17], the characteristics and the critical role of circRNA in co-/posttranscriptional regulation have only been revealed recently [4]. Studies have shown that circRNAs regulate gene expression mainly by acting as microRNAs (miRNAs) sponge [4, 18, 19], regulator of translation [20], binding protein [5], and RNA transport [21]. The most well known circular RNA sponges so far are ciRS-7 and sex-determining region Y (SRY), targeting microRNA-7 (miR-7) and microRNA-138 (miR-138), respectively [4, 6]. Moreover, misregulation of circRNAs leads to abnormal cellular functions and growth defects. Differentially expressed circRNAs in a wide range of cancers may play an important role in cancer initiation and progression [12]. For example, Huang et al. [7] have shown that cir-ITCH expression is typically downregulated in CRC in comparison with paired adjacent tissue. And Li et al. [8] have revealed that hsa_circ_002059 is downregulated in GC cancer. According to previous publications, circRNAs are more stable than linear mRNA in RNA exonucleases. The increase or decrease in circRNAs expression levels in tumors compared with normal tissues may serve as a useful biomarker in tumor diagnosis and prognosis.

In this study, based on previous research and two circRNA databases (circ2traits and CircBase), we found that the expression level of hsa_circ_0001649 is significantly downregulated in GC tissues when compared to the PCHNTs (*P* < 0.01). The analysis between circRNA expression level and clinicopathological data demonstrated that the expression level of hsa_circ_0001649 was more significantly decreased in poor and undifferentiated tumors than in well differentiated ones (*P* = 0.039). This phenomenon indicates that hsa_circ_0001649 level may have a negative correlation with GC pathological differentiation. However, detailed molecular mechanisms of hsa_circ_0001649 involved in GC progression are still mysterious. We next estimated the diagnostic value of hsa_circ_0001649 in GC. A comparatively satisfactory result was obtained by using ROC curve analysis (the sensitivity and specificity were 0.711 and 0.816, respectively; the area under the curve was 0.834). Our preliminary results indicate that hsa_circ_0001649 expression level was downregulated in GC tissue sample compared with normal ones and has the potential to be used as a novel biomarker for GC with high degrees of accuracy, specificity, and sensitivity.

Recently, some articles reported that changes of circRNAs expression level in fluids paralleled other somatic tissues and are thought to be connected with certain cancers. Li et al. [8] examined the levels of hsa_circ_002059 in plasma samples between preoperative and postoperative gastric cancer patients. They found that circulating hsa_circ_002059 expression level was significantly upregulated after surgery. Memczak et al. [10] sequenced circRNAs in human peripheral whole blood and testified the reproducibility of the detection method of thousands of circRNAs in blood samples. They concluded that circRNAs could be used as biomarker molecules in standard clinical blood samples. Li et al. [11] examined serum exosome from patients with CRC and normal serum and found 67 circRNAs were missing and 257 new circRNA species in CRC. Enlightened by the above studies, we tested hsa_circ_0001649 expression level in 20 paired GC serum samples. By analyzing experimental data, we found that hsa_circ_0001649 expression level was significantly upregulated in GC serum samples after surgery (*P* < 0.01). However, our study just validated the dysregulation of hsa_circ_0001649 in GC tissue and serum samples. Further experiments still need to be done to elucidate the role of hsa_circ_0001649 in the generation and progression of GC.

In summary, by comparing the expression level of hsa_circ_0001649 in tissue and serum samples, we found that detecting hsa_circ_0001649 between GC and normal ones has a relatively high sensitivity and specificity and, therefore, may be used as a biomarker for noninvasive screening of GC.

## Figures and Tables

**Table 1:** qRT-PCR primer sequences.

Primer set	Forward primer	Reverse primer
Hsa_circ_0001649	AATGCTGAAAACTGCTGAGAGAA	TTGAGAAAACGAGTGCTTTGG
GAPDH	TCGACAGTCAGCCGCATCTTCTTT	ACCAAATCCGTTGACTCCGACCTT

**Table 2:** Correlation between hsa_circ_0001649 expression and clinicopathological parameters in GC patients.

Parameters	Number of cases	Mean ± SD	*P* value
Age			
<60	42	0.14 ± 0.13	0.549^a^
≥60	34	0.16 ± 0.12	
Gender			
Male	61	0.15 ± 0.12	0.834^a^
Female	15	0.15 ± 0.16	
Pathological differentiation			
Well + moderate	31	0.18 ± 0.14	0.039^a^
Poor + undifferentiation	45	0.12 ± 0.10	
Depth of tumor invasion			
Tis, T1a, T1b	8	0.15 ± 0.16	0.366^a^
T2	10	0.08 ± 0.06	
T3	10	0.16 ± 0.17	
T4a, T4b	48	0.16 ± 0.17	
Lymph node metastasis			
N0	28	0.12 ± 0.11	0.389^a^
N1	23	0.18 ± 0.13	
N2	9	0.19 ± 01.8	
N3a, N3b	16	0.14 ± 0.09	
TNM Stage			
I, II	39	0.14 ± 0.13	0.386^a^
III, IV	37	0.16 ± 0.12	
CEA			
Positive	17	0.15 ± 0.11	0.914^a^
Negative	59	0.15 ± 0.13	
CA19-9			
Positive	10	0.15 ± 0.12	0.958^a^
Negative	66	0.15 ± 0.13	
CA-724			
Positive	8	0.08 ± 0.05	0.118^a^
Negative	68	0.16 ± 0.13	

^a^Using chi-square for this statistic.

## References

[1] Fitzmaurice C., Dicker D., Pain A. (2015). The global burden of cancer 2013. *JAMA Oncology*.

[2] Nashimoto A., Akazawa K., Isobe Y. (2013). Gastric cancer treated in 2002 in Japan: 2009 annual report of the JGCA nationwide registry. *Gastric Cancer*.

[3] Chen L.-L., Yang L. (2015). Regulation of circRNA biogenesis. *RNA Biology*.

[4] Memczak S., Jens M., Elefsinioti A. (2013). Circular RNAs are a large class of animal RNAs with regulatory potency. *Nature*.

[5] Jeck W. R., Sorrentino J. A., Wang K. (2013). Circular RNAs are abundant, conserved, and associated with ALU repeats. *RNA*.

[6] Hansen T. B., Jensen T. I., Clausen B. H. (2013). Natural RNA circles function as efficient microRNA sponges. *Nature*.

[7] Huang G., Zhu H., Shi Y., Wu W., Cai H., Chen X. (2015). Cir-ITCH plays an inhibitory role in colorectal cancer by regulating the Wnt/*β*-Catenin Pathway. *PLoS ONE*.

[8] Li P., Chen S., Chen H. (2015). Using circular RNA as a novel type of biomarker in the screening of gastric cancer. *Clinica Chimica Acta*.

[9] Qin M., Liu G., Huo X. (2016). Hsa-circ-0001649: a circular RNA and potential novel biomarker for hepatocellular carcinoma. *Cancer Biomarkers*.

[10] Memczak S., Papavasileiou P., Peters O., Rajewsky N. (2015). Identification and characterization of circular RNAs as a new class of putative biomarkers in human blood. *PLoS ONE*.

[11] Li Y., Zheng Q., Bao C. (2015). Circular RNA is enriched and stable in exosomes: a promising biomarker for cancer diagnosis. *Cell Research*.

[12] Chen Y., Li C., Tan C., Liu X. (2016). Circular RNAs: a new frontier in the study of human diseases. *Journal of Medical Genetics*.

[13] Sood R., Makalowska I., Galdzicki M. (2003). Cloning and characterization of a novel gene, SHPRH, encoding a conserved putative protein with SNF2/helicase and PHD-finger domains from the 6q24 region. *Genomics*.

[14] Qu Y., Gharbi N., Yuan X. (2016). Axitinib blocks Wnt/*β*-catenin signaling and directs asymmetric cell division in cancer. *Proceedings of the National Academy of Sciences of the United States of America*.

[15] Zaphiropoulos P. G. (1997). Exon skipping and circular RNA formation in transcripts of the human cytochrome P-450 2C18 gene in epidermis and of the rat androgen binding protein gene in testis. *Molecular and Cellular Biology*.

[16] Burd C. E., Jeck W. R., Liu Y., Sanoff H. K., Wang Z., Sharpless N. E. (2010). Expression of linear and novel circular forms of an INK4/ARF-associated non-coding RNA correlates with atherosclerosis risk. *PLOS genetics*.

[17] Capel B., Swain A., Nicolis S. (1993). Circular transcripts of the testis-determining gene Sry in adult mouse testis. *Cell*.

[18] Hansen T. B., Kjems J., Damgaard C. K. (2013). Circular RNA and miR-7 in cancer. *Cancer Research*.

[19] Sumazin P., Yang X., Chiu H.-S. (2011). An extensive MicroRNA-mediated network of RNA-RNA interactions regulates established oncogenic pathways in glioblastoma. *Cell*.

[20] Zhang Y., Zhang X.-O., Chen T. (2013). Circular Intronic Long Noncoding RNAs. *Molecular Cell*.

[21] Ashwal-Fluss R., Meyer M., Pamudurti N. R. (2014). CircRNA biogenesis competes with Pre-mRNA splicing. *Molecular Cell*.

